# Development of Novel Dual-Electrochemical Techniques for Capillary Electrophoresis by Means of Dead-Volume Free Flow Splitting

**DOI:** 10.1021/acs.analchem.6c04320

**Published:** 2026-09-11

**Authors:** Martin Koall, Johannes Weidinger, Frank-Michael Matysik

**Affiliations:** Institute of Analytical Chemistry, Chemo- and Biosensors, 9147 University of Regensburg, Universitätsstraße 31, 93053 Regensburg, Germany

## Abstract

The combination of capillary electrophoresis (CE) and electrochemistry, commonly in the form of amperometric detection (AD), has gained increasing importance by providing good selectivity, stability, and detection performance, especially when combined with non-aqueous media (NACE-AD). Despite these advantages, conventional NACE-AD systems face limitations when analyzing complex samples, being confronted with co-migrating analytes or the need for oxidative and reductive information at the same time. Established techniques for simultaneous oxidative and reductive detection often rely on complex electrode arrays, which are not able to provide completely independent electrode processes while posing complex electrochemical systems. To address these issues, a dual-detection concept (DDC) relying on dead-volume free flow splitting combined with two independent AD cells (CE-AD^2^) could provide a user-friendly alternative to existing approaches, avoiding most of these problems and giving new opportunities for method development. Moreover, dead-volume free flow splitting also allows other destructive, electroanalytical methods to be combined with CE-AD, e.g., voltammetry. The combination with linear-sweep voltammetry (LSV) offers a versatile tool for online identification of various analytes by their characteristic voltammogram (qualitative information) while using AD for quantitative information (CE-AD/LSV). This work presents two novel electrochemical DDCs for NACE using newly designed, synchronized, independent AD cells connected via a commercially available flow splitter. The first DDC, CE-AD^2^, simultaneously detects oxidizable and reducible species using independently optimized electrodes (platinum for oxidation and silver for reduction) and detection potentials within a single measurement run. The second approach, CE-AD/LSV, combines sensitive fixed-potential AD for quantification with LSV for online analyte identification. Together, these systems could improve flexibility, selectivity, and analytical performance for monitoring complex samples in NACE applications.

## Graphical abstract

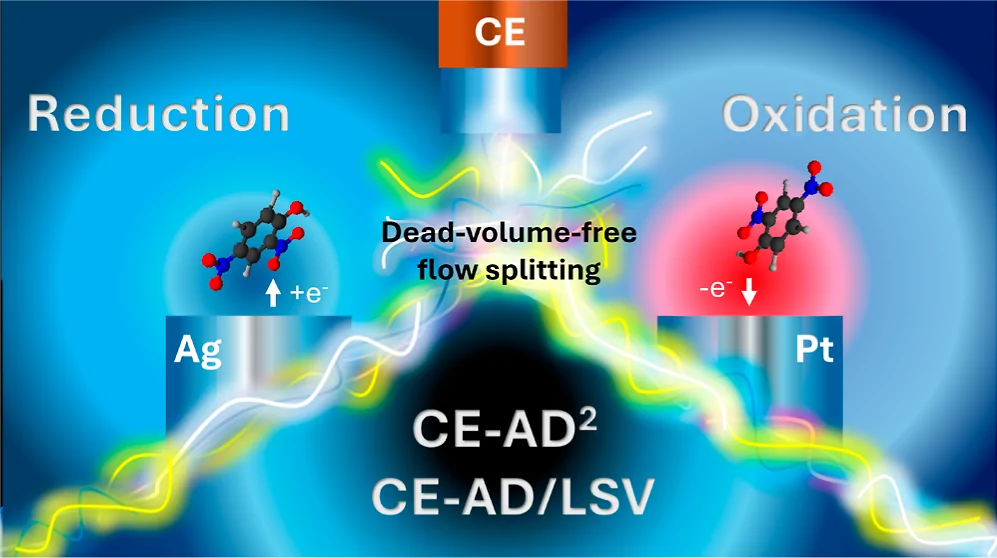

## 1 Introduction

An attractive mode of capillary electrophoresis (CE) is non-aqueous capillary electrophoresis (NACE), offering low electrophoretic currents and good long-term stability.^1–5^ NACE has been deployed in combination with conductivity,^6–8^ mass spectrometric,^8–10^ and UV^11,12^ detection in pharmaceutical and environmental contexts. Moreover, electrochemical methods play an ever-increasing role in the field of CE, commonly in the form of amperometric detection (AD), with ongoing developments including the use of nanomaterials as electrodes,^13^ novel methods in neurotransmitter analysis,^14^ or methods for quality control in the pharmaceutical^15^ and food industry.^16^ CE-AD shows improved sensitivity, selectivity, and stability, aided by the use of organic solvents that reduce noise due to low electrophoretic currents and enhance detection performance^4,17^ compared to aqueous systems. In the past, most NACE-AD applications were based on oxidative detection at platinum (Pt) electrodes. For example, nicotine in tobacco,^4^ trimetazidine in urine,^18^ nitenpyram in pesticide pills,^19^ and nicarbazin in poultry feed^20^ have been successfully determined using oxidative AD in combination with acetonitrile (ACN) based background electrolytes (BGEs). Recently, we were able to provide insight into reductive AD of nitrophenols at silver (Ag) electrodes in similar BGE systems.^21^

Electrochemical methods show high potential towards miniaturization, cost-efficiency, good sensitivity, and selectivity, given by the electrochemical properties of the analytes. However, standard CE-AD setups can struggle when it comes to complex samples, since the specific properties of the analytes have to match detection conditions, and co-migrating species cannot be distinguished. Furthermore, migration times for some species could differ significantly due to matrix effects in complex biological samples between the measurement runs, making a sequential application of different detection conditions (e.g., oxidation followed by reduction) challenging. The combination of oxidative and reductive detection within one measurement has a long tradition in the development of electroanalytical instrumentation. Multi-electrode configurations for electrochemical detection have been reported originally for liquid chromatography,^22^ followed by flow injection analyses,^23,24^ and aqueous CE, whereby various types of electrode arrays have gained great popularity. Geometries range from parallel dual-disc,^24,25^ or ring-disc configurations^25,26^ to complex multi-electrode arrays in the form of micro bands,^27,28^ disc arrays,^26^ or micro fiber bundles^29^ with various possible options for positioning in front of the capillary outlet.^30^

However, the electrode array introduces a complex electrochemical system in which oxidation and reduction cannot be optimized independently. While the linkage between oxidation and reduction in an electrode array can be advantageous for certain applications, such as generator–collector experiments,^28,31^ phenomena like redox cycling and feedback effects^32^ could influence the measurement significantly. A possible strategy to overcome these obstacles to ensure optimum oxidative and reductive detection could be the spatial separation of oxidative and reductive electrochemistry into two different detectors, exploiting dead-volume free flow splitting,^33^ which has been a powerful tool in the introduction of a novel dual-detection concept (DDC) based on AD and mass spectrometry.^34^ Here, two destructive detection principles were combined successfully using a commercially available flow splitter, yielding superior selectivity compared to CE-AD. The same principle could be applied to NACE combined with two independent AD cells operated at different detection conditions (CE-AD^2^). The application of CE-AD^2^ could be beneficial due to enhanced information gain in one measurement run, elimination of shifts in migration time between oxidative and reductive CE-AD runs, free choice of electrode material, and detection potential for oxidation and reduction, and the possibilities for electrochemical fingerprinting by simultaneous oxidative and reductive response of the analytes.

Another useful DDC based on this configuration could be the combination of AD and voltammetry. Starting with the pioneering work of O’Dea and Osteryoung^35^ in liquid chromatography combined with fast scan-square wave voltammetry, several approaches for combining flow systems, including CE, with voltammetry have been reported. Mainly, cyclic voltammetry and square wave voltammetry have been combined with CE over the past decades.^36–41^ In most cases, the electropherogram was generated from the voltammetric measurements, which were repeated periodically throughout a CE run. Here, the detection performance was strongly dependent on the voltammetric response and fast sampling times (μs-range). This could lead to worse sensitivity and stability compared to AD at a constant potential.^38^ The use of two independent, synchronized systems could exploit the high sensitivity and stability of fixed-potential AD while allowing for electrochemical identification of the analytes by online voltammetry at any point in the CE run, without compromising AD performance. Linear-sweep voltammetry (LSV) was used in combination with CE-AD (CE-AD/LSV) as it is an efficient and straightforward tool to characterize analytes regarding their electrochemical properties. Furthermore, the optimum AD potential under CE conditions could be determined in a single measurement, since the potential shift due to the high voltage also applies to online-LSV.

In this work, a newly developed, user-friendly AD cell design based on a linear capillary coupler and a commercially available flow splitter served as the centerpieces of CE-AD^2^ and CE-AD/LSV. Two identical AD cells (oxidation at Pt microelectrodes) and ferrocene derivatives as a model system were used to investigate cell synchronization, as well as for the investigation of CE-AD/LSV. For the investigation of CE-AD^2^, one Pt electrode was replaced with an Ag electrode for optimum reductive CE-AD, in conjunction with a BGE based on ammonium acetate and acetic acid.^21^ CE-AD^2^ showed good reproducibility and limits of detection (LODs) in the lower μM-range for a model system based on triethanol amine (TEA), trimethazidine (TMZ), 4-nitroaminobenzene (NAB), 4,4′-dinitrocarbanilide (DNC), and 2,4-dinitrophenol (DNP). This project aims to extend the application of CE-AD towards new DDCs solely based on NACE and electrochemistry, with opportunities for efficient oxidative and reductive monitoring of various compound classes in complex samples, while providing a high degree of freedom during method development.

## 2 Experimental Section

### 2.1 Reagents and Materials

The following chemicals were used, all of analytical grade: 4,4′-dinitrocarbanilide (DNC), 2,4-dinitrophenol (DNP), triethanolamine (TEA), trimetazidine (TMZ), and 4-nitroaminobenzene (NAB) were purchased from Sigma-Aldrich (St. Louis, USA). Acetonitrile (ACN), ammonium acetate, methanol (MeOH), and 0.1 M sodium hydroxide solution were purchased from Merck (Darmstadt, Germany). Acetic acid was purchased from Carl Roth (Karlsruhe, Germany). Ultrapure water was provided by a Milli-Q Advantage A10 system (Merck Darmstadt, Germany). Ferrocenemethanol (FcMeOH) was purchased from ABCR (Karlsruhe, Germany). (2-methyl-1-ferrocecnylmethyl)­trimethylammonium iodide ([FcMTMA]­I) was supplied by Strem Chemicals (Newburyport, USA).

All measurements were carried out using a background electrolyte consisting of 1 M acetic acid and 10 mM ammonium acetate in ACN/MeOH (70:30, v/v), because TMZ and DNC showed poor solubility in a BGE solely based on ACN.

Fused silica capillaries (75 μm inner diameter, 365 μm outer diameter, polyimide coated) were purchased from Polymicro Technologies (Phoenix, USA). The flow splitters FS CapTite Interconnect LS-C360-203-075 with 75 μm inner diameter one-piece fittings C360-100 were supplied by LabSmith (Livermore, USA). For the preparation of the AD electrodes, 25 μm Ag and Pt wires provided by Advent Research Materials (Oxford, United Kingdom) and 25 μm Au wires supplied by Goodfellow (Cambridge, United Kingdom) were used.

### 2.2 Novel Cell Design for Amperometric Detection

The novel AD cell design (see Figure 1) evolved from the earlier model, which is described in^4^ in detail. The new cell layout is aimed at providing better user-friendliness during assembly and better mechanical stability by the use of a modified linear capillary coupler (Besta, Wilhelmsfeld, Germany) instead of two symmetrically arranged stainless steel tubes for alignment. The coupler was housed in a laboratory-crafted AD cell body made from PTFE, which was filled with BGE during AD operation. The separation capillary was held in place by a micro-tight fitting from Besta (Wilhelmsfeld, Germany) inside the capillary coupler, while the laboratory-crafted working electrode (WE, 25 μm metal disc electrodes, manufacturing described below) was inserted into the capillary coupler, analogous to a micro-tight fitting. A cavity in the middle of the coupler enabled physical contact between the AD cell volume and the separation capillary, and WE to close the CE and AD circuits. A Pt wire, embedded in the cell body, served as the CE ground as well as the auxiliary electrode (AE). The reference electrode (RE), part of a PTFE cell cap, was based on a glass tube with an integrated diaphragm filled with separation BGE and an Ag/AgCl wire. The WE-to-capillary distance could be optimized using a digital microscope for optical control to approximately 75 μm (capillary inner diameter) for both AD cells in CE-AD^2^ and CE-AD/LSV.

**Figure 1:**
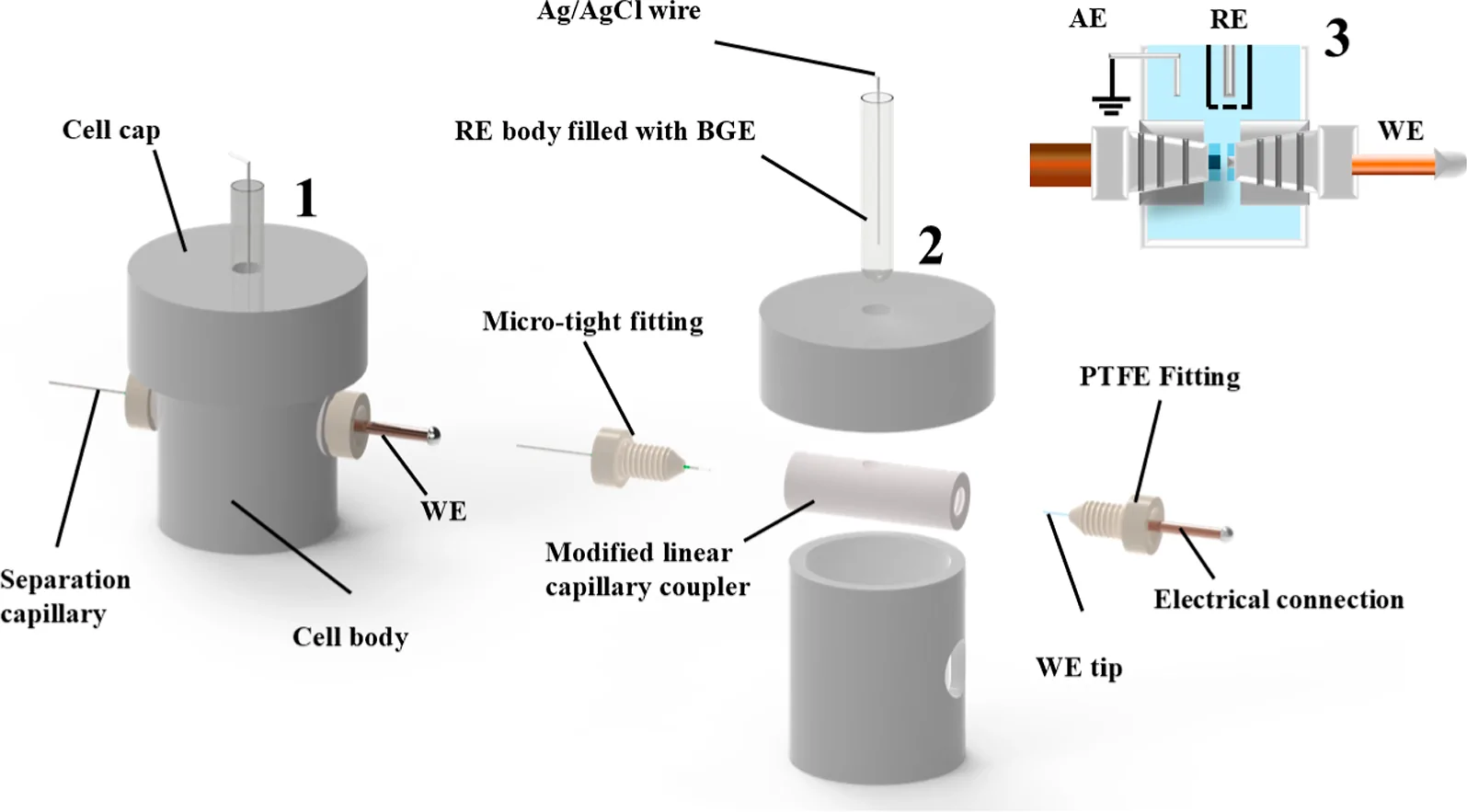
Novel design for an AD cell for CE. (1) Full 3D model of the cell. (2) Explosion model of the cell. (3) Schematic representation of the details of the configuration in cross-sectional view. The AD cell was filled with separation BGE during operation.

### 2.3 Microelectrode Fabrication

The microelectrodes were fabricated using the protocol described in^18^ Briefly, Ag and Pt micro wires were sealed in soda glass capillaries and integrated into laboratory-crafted PTFE fittings, here based on micro-tight fittings from Besta (Wilhelmsfeld, Germany). An electrical connection was established by a copper tube glued to the metal wire using silver epoxy.

### 2.4 Capillary Electrophoresis with Amperometric Detection

A custom-built, LabVIEW-controlled CE-system^19^ was coupled to an in-house constructed AD cell, which is described in Section 2.1. Electrode alignment was performed under an UltraZoom Pro digital microscope (dnt, Dietzenbach, Germany). The AD cells were enclosed in independent custom-built Faraday cages to minimize electromagnetic interference. The three-electrode assemblies were connected to a BioLogic SP-300 dual-channel potentiostat equipped with independent ultralow current modules (BioLogic, Seyssinet-Pariset, France) and operated using EC-Lab V11.62 software. The potentiostat was operated in floating mode to connect the CE grounding to the auxiliary electrode of the AD circuit. The sample was injected for 5 s (CE-AD and CE-AD^2^) and for 40 s (CE-AD/LSV) by a syphon effect due to a difference in height between the capillary inlet and outlet of 7 cm. The separation medium consisted of ACN, methanol, acetic acid, and ammonium acetate, and was operated at a comparatively low separation voltage, resulting in a reduced electroosmotic flow (EOF) compared with many aqueous separation configurations. The additional pressure-driven flow provided by the height difference facilitated the transport of highly negatively charged analytes, such as DNP under the investigated conditions, towards the detectors. A schematic representation of the setup can be seen in Figure 2, left side.

**Figure 2:**
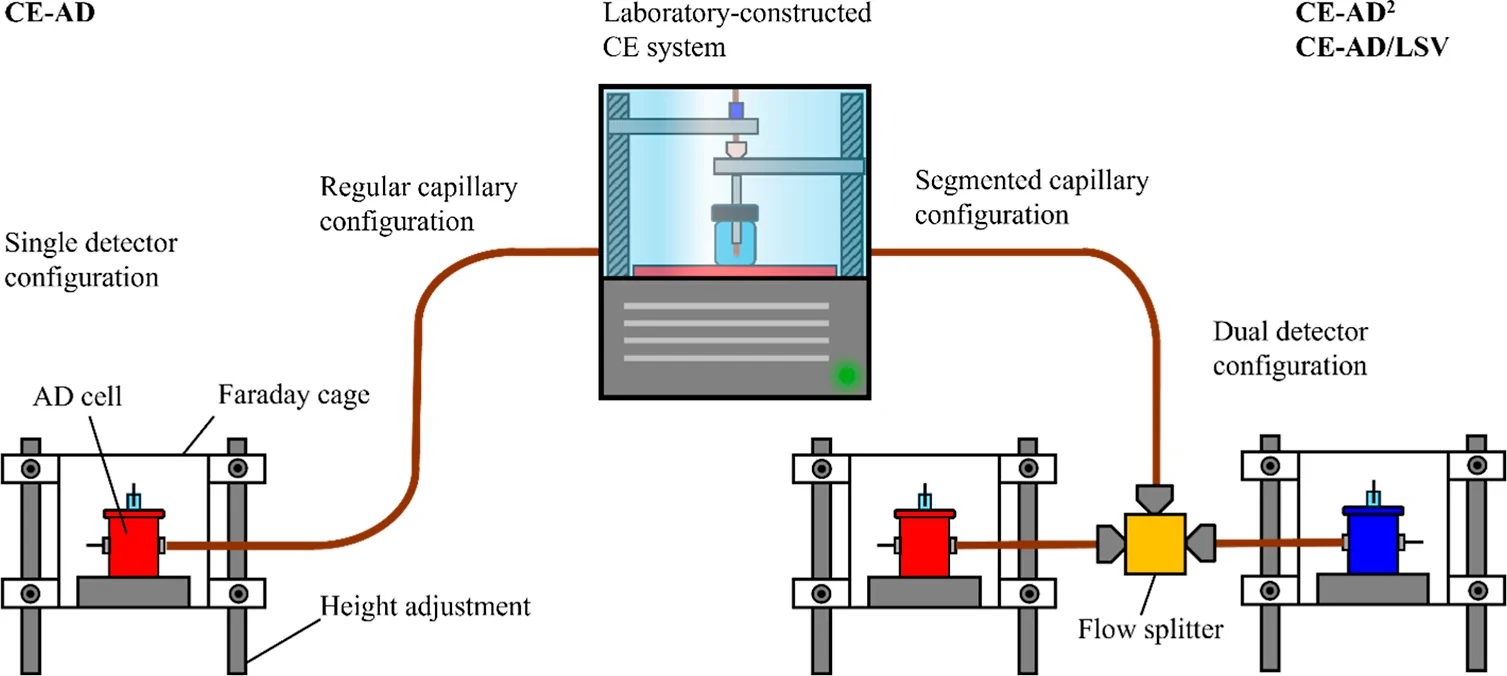
Schematic representation of different CE-AD setups. (Left) Standard CE-AD. (Right) Combining two independent amperometric detector cells with a flow splitter for achieving CE-AD^2^ and CE-AD/LSV. Center: laboratory-constructed CE system, which is used in all CE-AD and DDC approaches. A photograph can be found in Figure S. 1.

The separation capillary (length, 60 cm; I.D., 75 μm) was prepared according to the following procedure: the capillary ends were polished at a 90° angle. For conditioning, the capillary was sequentially rinsed with 0.1 M NaOH (10 min), deionized water (5 min), and background electrolyte (BGE: 10 mM ammonium acetate and 1 M acetic acid in ACN/MeOH (70:30,v/v), 30 min). The conditioning procedure was repeated at the beginning of each measurement day. A separation voltage of 10 kV was chosen for all measurements to allow a simultaneous recording of protonated (TEA, TMZ) and deprotonated species (DNC, DNP) with good separation performance, as higher separation voltages would lead to increased electrophoretic current and corresponding Joule heating and peak broadening.

### 2.5 Combining Two Independent Amperometric Detector Cells by Flow Splitting

For CE-AD^2^ and CE-AD/LSV, a similar setup to CE-AD was used. The CE device and potentiostat were used in the same way, whereby a segmented capillary configuration was deployed. One inlet capillary (length 30 cm before flow splitting; I.D., 75 μm) and two capillaries at the outlet end (length 30 cm after flow splitting; I.D., 75 μm) were connected by a T-shaped flow splitter (I. D. 75 μm). At the two parallel outlet ends, two identical AD cells (see Section 2.2) were installed and housed in two individual Faraday cages to prevent electromagnetic interference. The height of the Faraday cages could be adjusted separately. Adjusting the height difference between the detectors (while both detectors were placed lower in height than the capillary inlet) allowed synchronization of the migration times of the analytes after flow splitting. This approach was originally reported in the context of a DDC based on AD and MS.^34^ A schematic representation of the setup is depicted in Figure 2, right side, and a photograph can be found in Figure S. 1. For CE-AD/LSV, the two detectors were equipped with 25 μm Pt-disc electrodes. Here, one channel of the SP 300 potentiostat was constantly operated in chronoamperometric mode, while at the other, an LSV measurement was performed when a peak in AD was measured, resulting in the electrochemical fingerprint of the AD signal. For CE-AD^2^, one Pt electrode was changed to an Ag electrode with the same geometry. Both cells were operated in chronoamperometry mode (oxidation and reduction, respectively), giving oxidative and reductive information simultaneously. The migration times of the analytes in the two channels were synchronized by optimizing the height difference between the two detector cells. The potential shift due to high voltage had to be determined for each cell individually by cyclic voltammetry at the beginning of the measurement day and compensated for oxidation^4^ and reduction,^21^ respectively.

### 2.6 Voltammetric Characterization of the Model Systems

Offline LSV was used for the characterization of the model system based on a 25 μm Pt disc for oxidative detection and a 25 μm Ag disc electrode for reductive detection was used in combination with a 4 mL batch cell. An Ag/AgCl wire served as a reference electrode, and a Pt wire was used as a counter electrode. LSV measurements were performed from 0.2 to 1.4 V for oxidation and from −0.2 to −1.4 V for reduction at a scan rate of 100 mV/s. Separation BGE (10 mM ammonium acetate and 1 M acetic acid in ACN/MeOH 70:30, v/v) and 400 μM solutions of TEA, TMZ, NAB, and TNP were investigated oxidatively, and TMZ, NAB, DNC, and DNP were investigated reductively.

## 3 Results and Discussion

### 3.1 Combining Two Independent Amperometric Detector Cells by Flow Splitting

CE-AD^2^ and CE-AD/LSV rely on two independent, synchronized AD cells. It is of central importance to ensure the simultaneous detection of the same analyte in both detectors. For the synchronization of the migration time, a similar approach to CE-AD/MS^34^ was chosen. In this work, the height difference of the two AD cells was optimized using [FcMTMA]­I as a model analyte and oxidation at a Pt electrode at 0.8 V (see Figure S. 2). For no height difference, [FcMTMA]­I could be detected simultaneously in both cells (CH1 and CH2), due to an identical detector layout. The following CE-AD/LSV and CE-AD^2^ measurements were carried out using an inter-detector no height difference. It has to be mentioned that this value could differ significantly when detectors with varying geometries are deployed.

### 3.2 Dual Amperometric Detection (Oxidation/Reduction)

The first DDC, namely CE-AD^2^, combined individual synchronized AD cells for simultaneous oxidative and reductive detection, while providing maximum freedom in optimizing oxidative and reductive conditions regarding electrode materials and detection potentials, to analyze complex samples efficiently. A model system based on TEA, TMZ, NAB, DNC, and DNP was used in conjunction with Pt electrodes for oxidation and Ag electrodes for reduction.

### 3.3 Electrochemical Characterization of the Model System for CE-AD^2^

Initially, 400 μM solutions of TEA, TMZ, NAB, DNC, and DNP were investigated using offline LSV in the potential range from 0.2 to 1.4 V for electrochemical oxidation at Pt electrodes and from −0.2 to −1.4 V for reduction at Ag electrodes. The measurements displayed in Figure 3 show the electrochemical activity of TEA, TMZ, NAB, and DNP under oxidative conditions and of TMZ, NAB, DNC, and DNP under reductive conditions. The detection potentials for CE-AD^2^ were set to 1.2 V (oxidation) and −1.2 V (reduction) after the compensation of Δ*E*, as the respective analytes showed a distinct oxidative and reductive current compared to the BGE (10 mM ammonium acetate and 1 M acetic acid in ACN/MeOH (70:30, v/v)). The following CE-AD and CE-AD^2^ experiments were carried out using these conditions, which are not the optimum detection conditions for the single analytes, and therefore, other CE-AD methods could be more sensitive for some analytes.

**Figure 3:**
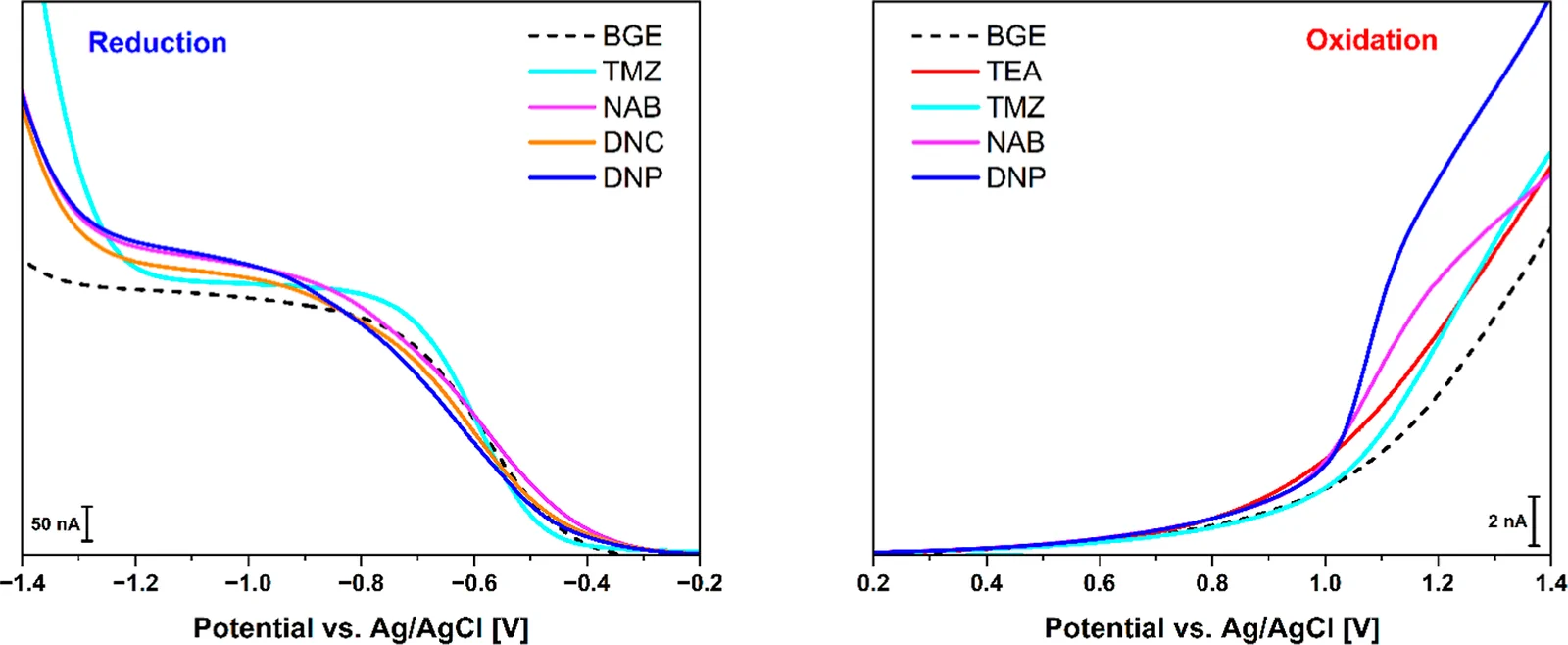
Offline LSV measurements of a BGE (10 mM ammonium acetate and 1 M acetic acid in ACN/MeOH 70:30, v/v, dashed line), and 400 μM of TEA (red), TMZ (cyan), NAB (purple), DNC (orange), and DNP (blue) in BGE. Oxidation from 0.2 to 1.4 V at a 25 μM Pt disc and reduction from −0.2 to −1.4 V at a 25 μM Pt disc electrode. Scan rate: 100 mV/s.

### 3.4 Analytical Performance of the Novel Dual Detection Concept CE-AD^2^

For the analytical characterization of the novel DDC CE-AD^2^, a mixture of TEA, TMZ, NAB, DNC, and DNP was used, whereby NAB was used as an EOF marker and to synchronize the migration times of the analytes, since it showed an oxidative and reductive AD response and could be used as a fixed point in the electropherogram. The respective electropherogram of a 400 μM mixture can be seen in Figure 4.

**Figure 4:**
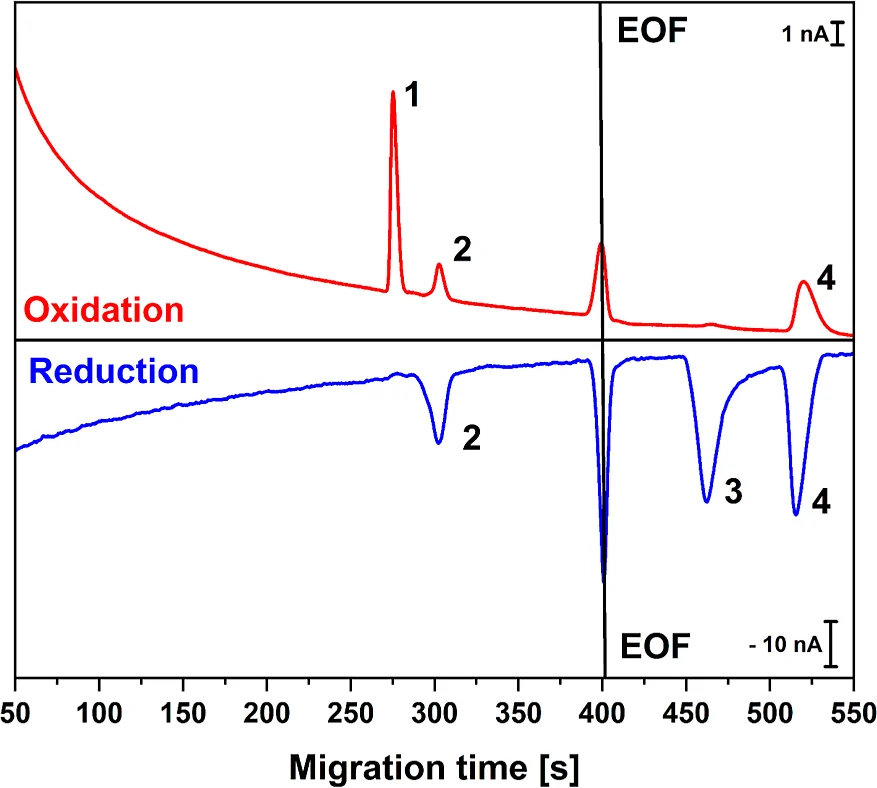
CE-AD^2^ measurement of a mixture containing 400 μM TEA (1), TMZ (2), NAB (EOF), DNC (3), and DNP (4) in BGE 10 mM ammonium acetate and 1 M acetic acid in ACN/MeOH (70:30, v/v). Oxidative detection was performed at 1.2 V (25 μm Pt disc) at CH1, and reductive detection was performed at −1.2 V (25 μm Ag disc) at CH2. The amperometric response of CH2 (reduction) was multiplied by the scaling factor of 0.2 to provide a simultaneous overview of oxidation and reduction current. CE separation at 10 kV, capillary I. D. 75 μm, capillary length: 30 cm (before flow splitting) and 30 cm after flow splitting, injection for 5 s at a height difference of 7 cm between inlet and outlet.

Here, TEA (1) showed an oxidative electrochemical response in the migration window for positively charged analytes (protonation) at 275 s, and TMZ (2) gave a smaller oxidative and reductive response at 302 s in the migration window for positively charged analytes (protonation). NAB migrated with the EOF at 405 s (neutral), showing oxidative and reductive activity. In the migration window for negatively charged analytes (deprotonation), DNC (3) with a migration time of 461 s could be detected reductively, and DNP (4) with a migration time of 515 s could be detected oxidatively and reductively. These results showed that some analytes could be detected in oxidative mode (e. g., TEA), others in reductive mode (e. g., DNC), and others in both modes (e. g., DNP). This difference in oxidative and reductive electrochemical behavior could be used to distinguish co-migrating analytes and provide certain possibilities for electrochemical fingerprinting regarding oxidation and reduction.

To characterize the analytical performance, the reproducibility of CE-AD^2^ in oxidative and reductive mode was investigated. The results regarding migration time, peak height, peak area, and full width at half maximum (FWHM) can be found in Table 1. All parameters investigated showed good reproducibility with low standard deviation, which is in good agreement with other CE-AD methods.^18,20,21^

**Table 1:** Reproducibility of the DDC CE-AD^2^ ^a^

signal	substance	migration time (s)	peak height (nA)	peak area (nAs)	FWHM (s)
DDC CE-AD^2^ (oxidation)					
1	TEA	275 ± 1.0	8.5 ± 0.8	38.7 ± 3.7	4.3 ± 0.1
2	TMZ	302 ± 1.0	1.3 ± 0.1	7.9 ± 0.5	5.7 ± 0.3
EOF	NAB	399 ± 1.0	3.2 ± 0.1	22.2 ± 3.1	6.9 ± 0.1
-	DNC	-	-	-	-
4	DNP	518 ± 1.7	2.1 ± 0.1	28.9 ± 1.3	13.0 ± 0.2
DDC CE-AD^2^ (reduction)					
-	TEA	-	-	-	-
2	TMZ	302 ± 1.0	–15.1 ± 0.1	–194 ± 12.4	11 ± 1.6
EOF	NAB	400 ± 1.0	–59.0 ± 0.6	–308 ± 13.2	4.5 ± 0.4
3	DNC	461 ± 1.0	–29.5 ± 0.2	–441 ± 32.7	13.2 ± 0.3
4	DNP	515 ± 1.0	–31.7 ± 0.1	–350 ± 12.8	10.8 ± 0.2

a Measurements of a 400 μM mixture of TEA, TMZ, NAB, DNC, and DNP in BGE (10 mM ammonium acetate and 1 M acetic acid in ACN/MeOH (70:30, v/v). Oxidative detection was performed at 1.2 V (25 μM Pt electrode) at CH1, and reductive detection was performed at −1.2 V (25 μM Ag electrode) at CH2. Migration time, peak height, peak area, and FWHM (mean and standard deviation) for each species (*n* = 10).

The next point to be addressed was to clarify the influence of flow-splitting on sensitivity. For this purpose, the LODs were determined for CE-AD under oxidative and reductive conditions separately, followed by CE-AD^2^. The results displayed in Table 2 show the same trend for all the analytes investigated. CE-AD and CE-AD^2^ showed low LODs in the lower μM range, making both concepts applicable in many analytical contexts. However, CE-AD^2^ showed higher LODs for all analytes tested (in contrast to other DDCs based on CE-AD^34^). In the present work, oxidative and reductive detection were investigated, leading to significantly longer migration times for all analytes for CE-AD^2^ compared to CE-AD. An exemplary comparison of an electropherogram of 400 μM DNP measured with oxidative and reductive CE-AD and the DDC CE-AD^2^ can be seen in Figure S. 3. Here, an increase in migration time, a decrease in peak height, and a peak broadening effect (indicated by the higher FWHM value) could be observed. This is a result of different residence times of analyte zones in CE-AD and CE-AD^2^, respectively. In addition, effects of different effective field strengths (in the individual capillary segments for the splitting mode) in CE-AD and CE-AD^2^ could lead to this observation, although the flow-splitting can be considered dead-volume free. Furthermore, in this project, precision-machined flow splitters were used. However, these parts can suffer from production-related micro-grating or small dimensional and angular deviations. This can also lead to some increased flow resistance.

**Table 2:** LODs for TEA, TMZ, and DNP at Oxidative Conditions (1.2 V at Pt) and TMZ, DNC, and DNP at Reductive Conditions (−1.2 V at Ag) in the Concentration Range from 20 to 60 μM for Oxidative and Reductive CE-AD and the DDC CE-AD^2^ for S/N = 3, *n* = 3^a^

	CE-AD	CE-AD	DDC CE-AD^2^	
substance	LOD (oxidation) (μM)	LOD (reduction) (μM)	LOD (oxidation) (μM)	LOD (reduction) (μM)
TEA	9.8 ± 0.1	-	19.3 ± 0.5	-
TMZ	14.0 ± 0.2	15.1 ± 0.3	43 ± 3.6	42 ± 1.8
NAB	EOF	EOF	EOF	EOF
DNC	-	8.2 ± 0.4	-	14.4 ± 0.7
DNP	3.8 ± 0.1	10.5 ± 0.3	7.3 ± 0.3	18.8 ± 0.8

a The sensitivities of determinations derived from the calibration functions can be found in Table. S. 1.

### 3.5 Combining Amperometric Detection and Voltammetry for Electrochemical Fingerprinting

The second DDC to be investigated was CE-AD/LSV. Here, the focus was on the oxidative detection at Pt electrodes, whereby [FcMTMA]I (cationic species) and FcMeOH (EOF marker) were used as a first model system. In Figure 5, a CE-AD/LSV measurement of a 400 μM mixture of [FcMTMA]I and FcMeOH is shown.

**Figure 5:**
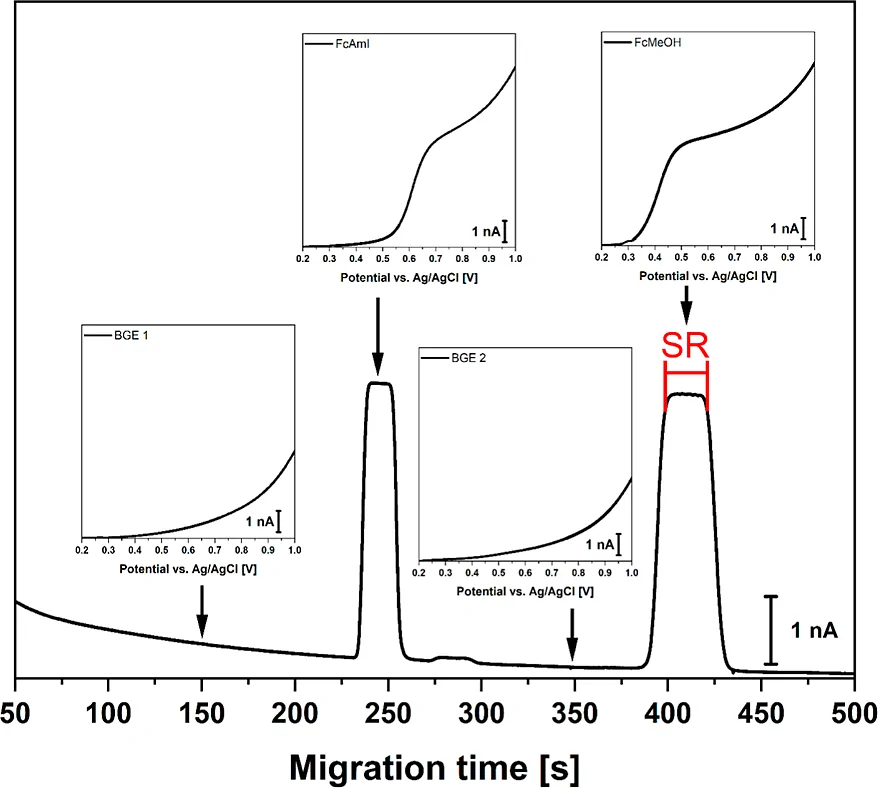
CE-AD/LSV measurement of a 400 μM mixture of [FcMTMA]I and FcMeOH in BGE (10 mM ammonium acetate and 1 mM acetic acid in ACN/MeOH 70:30, v/v). CE-AD at 0.8 V was performed at CH1, and online-LSV measurements (scan rate: 100 mV/s) of the [FcMTMA]I and FcMeOH signals and BGE (baseline) were performed at CH2 using identical 25 μm Pt working electrodes in both cells. Exemplary sampling region (SR) for analyte signal marked in red. CE separation at 10 kV, capillary I. D. 75 μm, capillary length: 30 cm (before flow splitting) and 30 cm after flow splitting, injection for 40 s at a height difference of 7 cm between inlet and outlet.

The electropherogram from continuous CE-AD shows two signals, which correspond to [FcMTMA]I at 240 s and FcMeOH at 415 s. Online-LSV measurements were performed at the positions marked in the electropherogram. The voltammogram of BGE (baseline) was measured before [FcMTMA]I was detected. The second voltammogram represents [FcMTMA]­I. The third (BGE, baseline for the second time) was performed after [FcMTMA]I detection and showed no difference to the first one, allowing cross-influence-free LSV measurements of the analyte bands after CE separation. The last voltammogram represents FcMeOH. [FcMTMA]I and FcMeOH could be identified by their characteristic voltammogram. However, it has to be noted that the LSV measurements were performed under CE-conditions (significant influence of high voltage). To be comparable with the offline LSV measurements, the potential shift (Δ*E*) has to be compensated. An exemplary comparison of offline LSV and CE-AD/LSV can be seen in Figure S. 4. Here, a positive separation voltage of 10 kV was applied, leading to the potential shift, which had to be compensated by subtracting the Δ*E* (here 100 mV). Δ*E* has to be determined at the start of the measurement session using cyclic voltammetry. After compensation, LSVs obtained in CE-AD/LSV and offline LSV showed the same onset and peak potentials. Moreover, CE-AD/LSV could also be time-saving, since the optimum detection potential under CE conditions could be determined within one measurement run, since AD and LSV experience the same Δ*E* due to high voltage, and no additional compensation has to be made when translating potentials of LSV into AD.

To effectively combine CE and voltammetry, the sampling region (SR) is of central importance. It is necessary to establish a concentration plateau in the AD trace during the LSV measurement to get reliable voltammograms. In CE-AD/LSV, it can be simply achieved by adjusting the LSV scan rate and/or the CE injection time. Here, the injection time was set to 40 s to allow a potential scan rate of 100 mV/s.

A CE-AD/LSV measurement of a more complex sample can be seen in Figure S. 5, which was based on 400 μM TEA, TMZ, NAB, and DNP, and was investigated under the same CE-AD/LSV conditions, whereby each AD signal showed a distinct, characteristic voltammetric signature.

## 4 Conclusion

In this study, we presented two powerful DDCs solely based on electrochemical methods. Dead-volume free flow splitting and a novel user-friendly AD cell design were of central importance to CE-AD^2^ and CE-AD/LSV.

The combination of independent, simultaneous oxidative and reductive CE-AD posed high flexibility in method development (choice of electrode material and detection potential), while giving the opportunity of electrochemical fingerprinting by combined oxidative and reductive detection and the option to distinguish co-migrating species, due to different detection conditions at both potentiostat channels. CE-AD^2^ showed good reproducibility and low LODs (in the μm-range) using a model system based on TEA, TMZ, NAB, DNC, and DNP, which are straightforward for many determinations, and the additional benefits gained by CE-AD^2^ could bring advantages compared to the application of a single CE-AD approach.

CE-AD/LSV enhances AD by the option to identify each compound in the electropherogram by its characteristic voltammogram, using [FcMTMA]­I and FcMeOH as a model system. Also, a more complex system based on TEA, TMZ, NAB, and DNP was investigated successfully.

These results demonstrated the opportunities presented by AD and voltammetry combined with NACE in DDCs based on independent AD cells, bringing many options for the routine analyses of complex samples, requiring high separation efficiency, low sample consumption, cost-efficiency, and adaptability. The results gained in NACE could also be translated easily into aqueous CE using suitable electrode materials (e. g., carbon fibers), due to the modular nature of the setup and the adaptability of the AD cells used for detection.

Furthermore, the modular nature of the setup and the independent AD cells allow easy implementation of other amperometric and voltammetric techniques, e.g., pulsed methods to achieve further DDCs based on electrochemistry.

## Supplementary Material



## Notes

Supporting Information Additional experimental details, analytical characterization, including photographs of experimental setup (PDF)

## Abbreviations

ACN: acetonitrile
AD: amperomentric detection
Ag: silver
CE: capillary electrophoresis
BGE: background electrolyte
CE-AD: capillary electrophoresis with amperometric detection
CE-AD^2^: capillary electrophoresis with independent amperometric detection at two detectors
CE-AD/LSV: capillary electrophoresis with amperometric detection and online voltammetry
DDC: dual-detection concept
DNC: 4,4′-dinitrocarbanilide
DNP: Dinitrophenol
FcMeOH: ferrocne methanol
[FcMTMA]­I: ferrocecnylmethyl)­trimethylammonium iodide
FWHM: full width at half maximum
I. D.: inner diameter
LOD: limit of detection
LSV: linear-sweep voltammogram
NAB: nitroaminobenzene
NACE: nonaqueous capillary electrophoresis
Pt: platinum
SR: sampling region
TEA: triethanolamine
TMZ: trimetazidine.
