# Supplementary material for: Development of Novel Dual-Electrochemical Techniques for Capillary Electrophoresis by Means of Dead-Volume Free Flow Splitting
Source: Anal Chem. 2026 Sep 11;98(37):27567–74. doi: 10.1021/acs.analchem.6c04320 (PMC13602846; doi:10.1021/acs.analchem.6c04320)
Supplement: Supplementary file 1 [file ac6c04320_si_001.pdf]

# Development of Novel Dual-Electrochemical Techniques for Capillary Electrophoresis by Means of Dead-Volume Free Flow Splitting

*Martin Koall, Johannes Weidinger, Frank-Michael Matysik**

Institute of Analytical Chemistry, Chemo- and Biosensors, University of Regensburg, Universitätsstraße 31, 93053 Regensburg, Germany).

**Supporting information**

**Table of Contents:**

**Fig. S. 1:** Photographs of the experimental setup

**Fig. S. 2:** CE-AD$^2$ measurements of 1 mM [FcMTMA]I for various height differences between the two amperometric detector cells

**Fig. S. 3:** Comparison of CE-AD and CE-AD$^2$ characteristics for oxidative and reductive detection

**Table S. 1.** Sensitivities for determinations of TEA, TMZ, and DNP (oxidative detection) and TMZ, DNC, and DNP (reductive detection) using CE-AD and CE-AD$^2$, respectively

**Fig. S. 4:** LSV of FcMeOH under CE flow conditions for CE-AD/LSV compared to offline LSV

**Fig. S. 5:** CE-AD/LSV measurements for a mixture of TEA, TMZ, NAB, and DNP

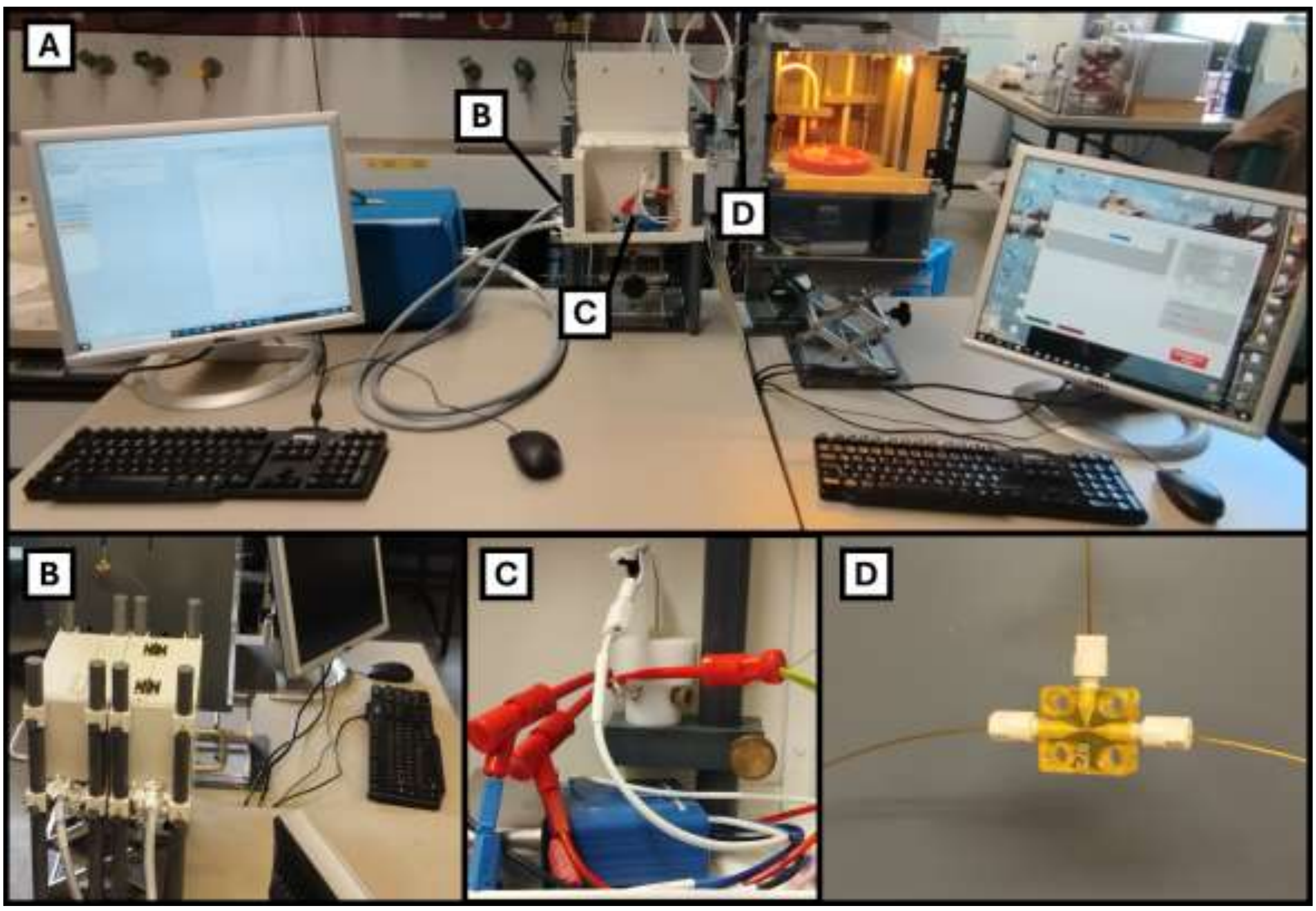


**Fig. S. 1:** Photographs of the CE setup used in CE-AD$^2$ and CE-AD/LSV. Overview of the setup (A), side view of two parallel, independent Faraday cages equipped with AD cells (B), close-up view of the AD cells mounted two Faraday cages (C), and close-up view of the T-shaped flow splitter (D).

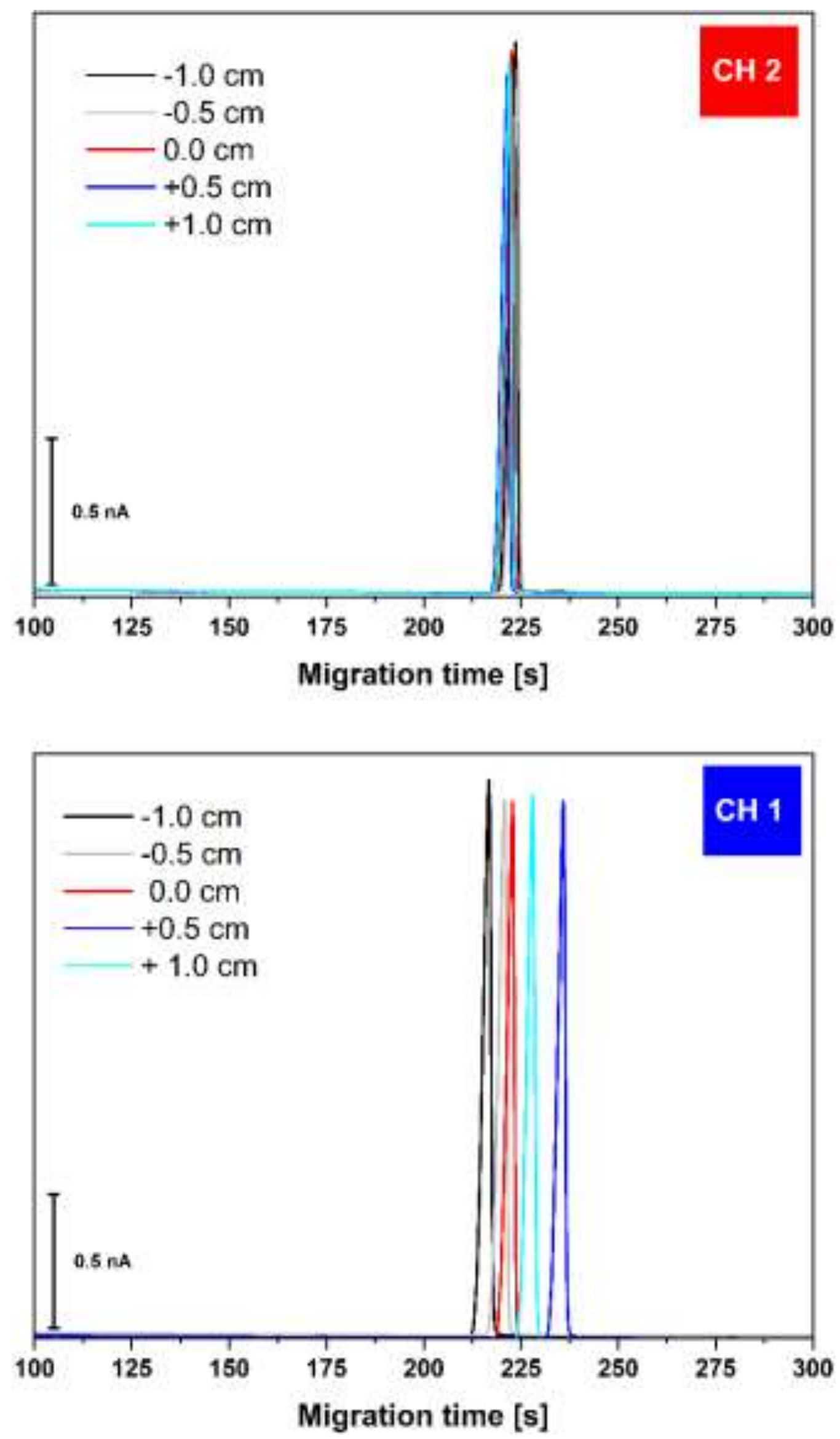


**Fig. S. 2:** CE-AD[2] measurements of 1 mM [FcMTMA]I in BGE (10 mM ammonium acetate and 1 M acetic acid in ACN:MeOH 70:30, v:v). Oxidation at 0.8 V was performed at both channels (CH1 and CH2). The height difference of CH1 and CH2 was optimized by adjusting the height of CH1. Synchronized migration time of [FcMTMA]I at 0.0 cm height difference of CH1 and CH2 (identical detectors).
CE separation at 10 kV, capillary I. D. 75 µm, capillary length: 30 cm (before flow splitting) and 30 cm after flow splitting, injection for 5 s at a height difference of 7 cm between inlet and outlets.

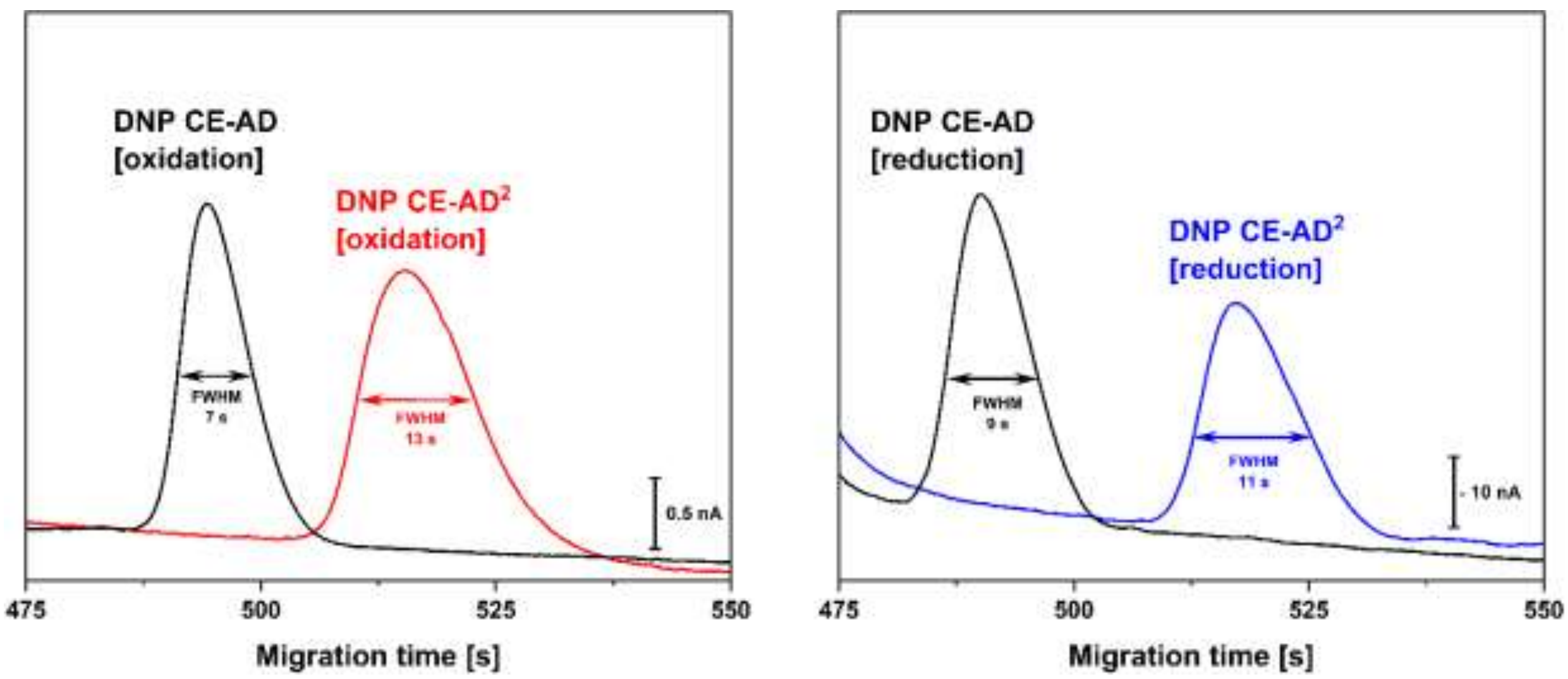


**Fig. S. 3:** Comparison of the signals for 400 µM DNC in BGE for the DDC NACE-$AD^2$ using a flow splitter (oxidation red, reduction blue) and CE-AD without flow splitting (black electropherograms). Oxidation at 1.2 V (left) and reduction at - 1.2 V (right). Peak broadening for oxidative and reductive peaks in the case of flow splitting is indicated by the higher full-width at half-maximum values (FWHMs) for the DDC, caused by different effective field strengths and flow resistance of CE-AD and CE-$AD^2$.
CE separation at 10 kV, injection for 5 s at a height difference of 7 cm between inlet and outlet.
DDC: Capillary I. D. 75 µm, length: 30 cm (before flow splitting) and 30 cm after flow splitting
CE-AD: Capillary I. D. 75 µm, length: 60 cm.

**Table S. 1**. Sensitivities (slope of the calibration function) for TEA, TMZ, and DNP at oxidative conditions (1.2 V at Pt) and TMZ, DNC, and DNP at reductive conditions (-1.2 V at Ag) in the concentration range from 20 to 60 µM for oxidative and reductive CE-AD and the DDC CE-$AD^2$

| Substance | CE-AD LOD (oxidation) [nA/µM] | CE-AD LOD (reduction) [nA/µM] | DDC CE-$AD^2$ LOD (oxidation) [nA/µM] | DDC CE-$AD^2$ LOD (reduction) [nA/µM] |
|---|---|---|---|---|
| TEA | 37 | - | 25 | - |
| TMZ | 23 | 23 | 4 | 5 |
| NAB | EOF | EOF | EOF | EOF |
| DNC | - | 21 | - | 132 |
| DNP | 98 | 28 | 39 | 111 |

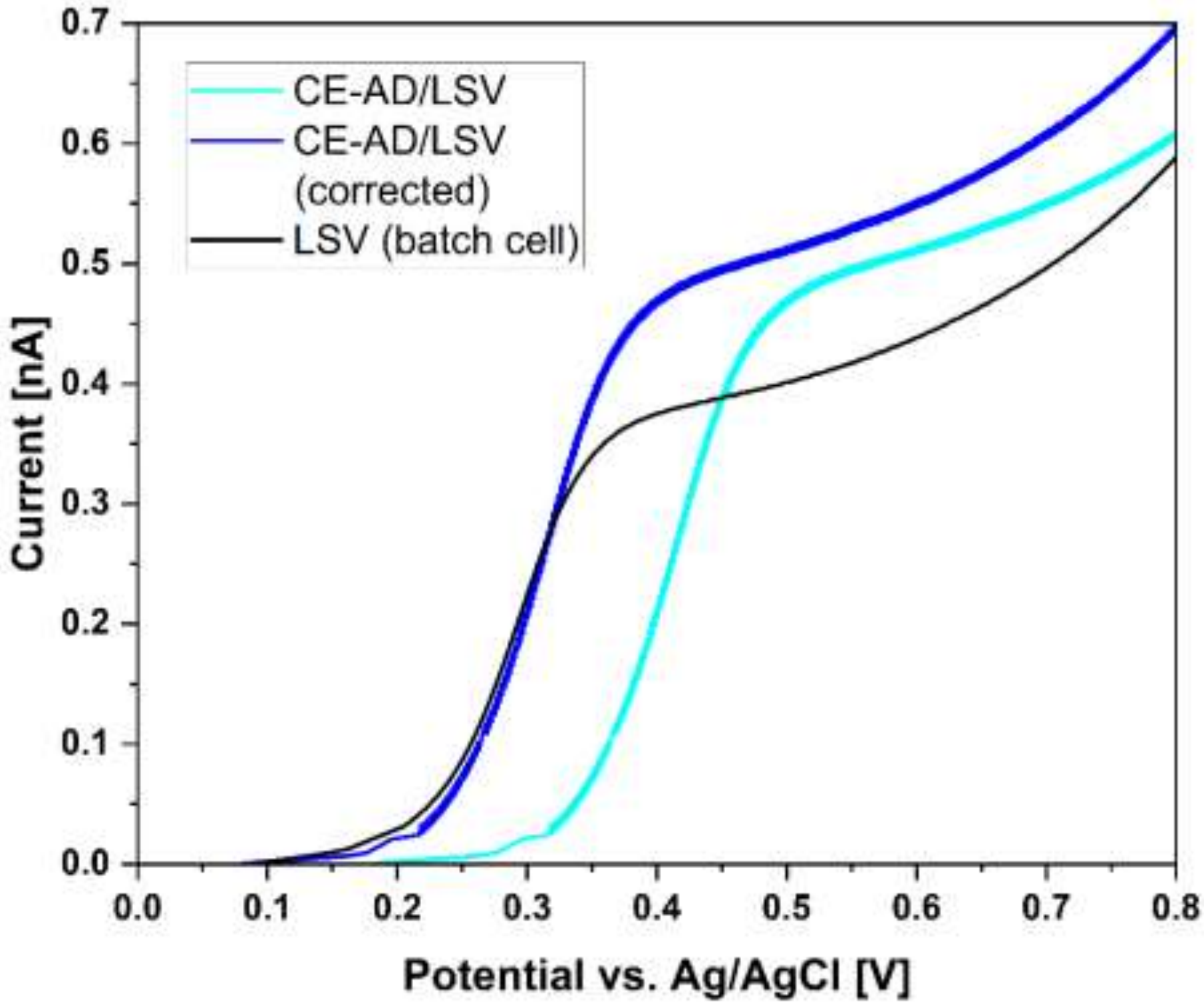


**Fig. S. 4**: Comparison of LSV measurements of 400 µM FcMeOH in BGE (10 mM ammonium acetate and 1 M acetic acid in ACN:MeOH (70:30, v:v). Voltammogram of CE-AD/LSV (cyan). Offline voltammogram using a batch cell (black) was added. Scan rate: 100 mV/s. CE-AD/LSV voltammogram with corrected potential shift (here 100 mV, blue voltammogram). The potential-shift was determined by cyclic voltammetry at the beginning of the measurement day.

CE-AD/LSV conditions were as in **Fig. 3**.

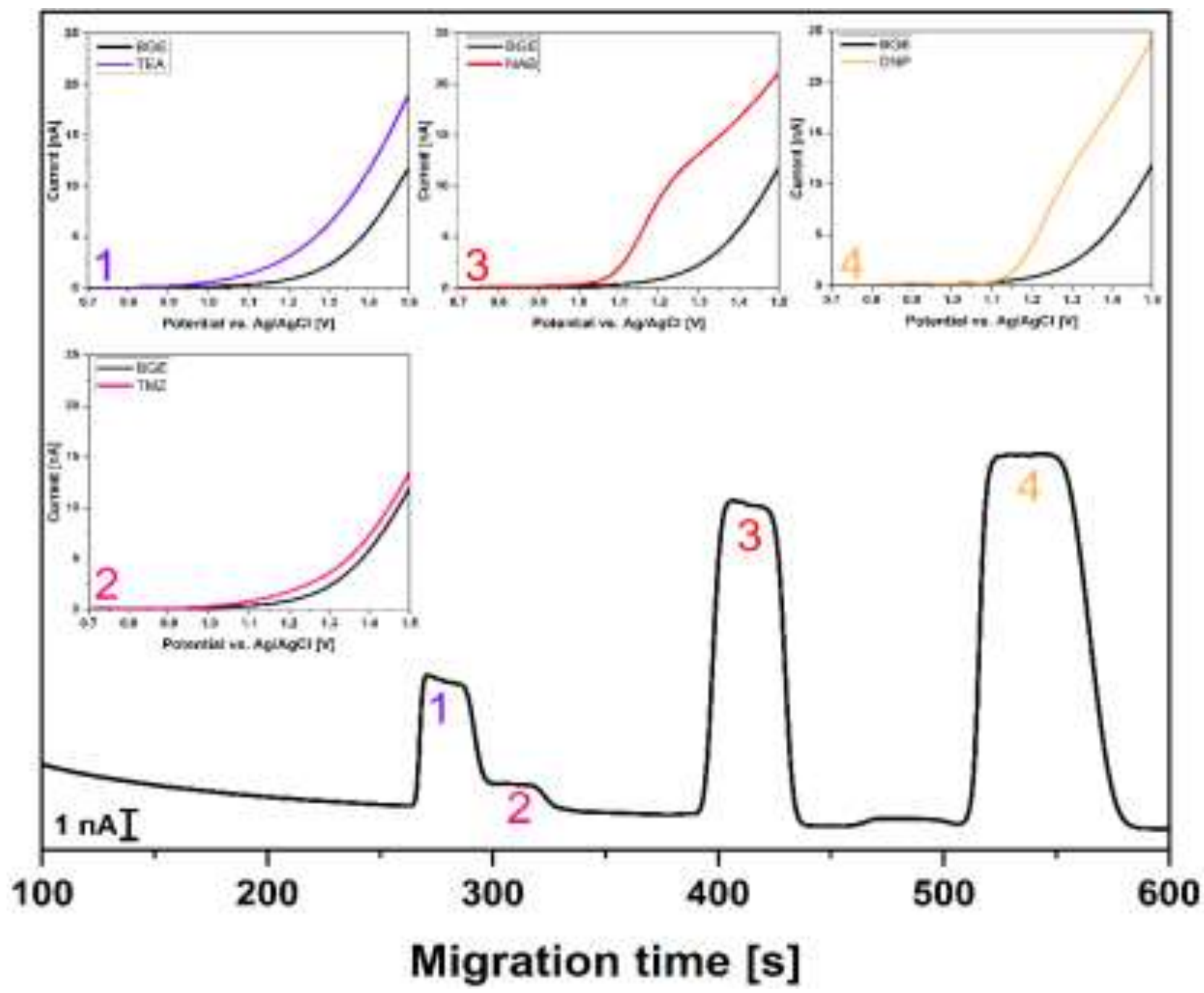


**Fig. S. 5:** CE-AD/LSV measurements of a mixture of 400 µM TEA, TMZ, NAB, and DNP in BGE (10 mM ammonium acetate and 1 mM acetic acid in ACN:MeOH 70:30, v:v). Oxidation at 1.2 V was performed at CH1, and online-LSV measurements (potential scan rate: 100 mV/s) of the TEA (1), TMZ

(2), NAB (3), and DNC (4) signals were performed at CH2 using identical 25 μm Pt working electrodes in both cells. A CE-AD/LSV voltammogram of BGE (black) (10 mM ammonium acetate and 1 mM acetic acid in ACN:MeOH (70:30, v:v)). CE separation at 10 kV, capillary I. D. 75 μm, capillary length: 30 cm (before flow splitting) and 30 cm after flow splitting, injection for 40 s at a height difference of 7 cm between inlet and outlet.
